# Integrating safety planning, problem-solving therapy and peer support for suicide prevention among adolescents living with HIV in Malawi: An application of the ADAPT-ITT adaptation framework

**DOI:** 10.1017/gmh.2026.10181

**Published:** 2026-05-18

**Authors:** Melissa Ann Stockton, Katherine Waddell, Nivedita L. Bhushan, Steven Mphonda, Victoria Constantine, Charles Masulani-Mwale, James January, Greg Brown, Michael Udedi, Jeromy Nyirenda, Ezekiel Mahuka, Brian Pence, Bradley Gaynes, Matthew T. K. Owusu, Ruth Verhey, Kazione Kulisewa

**Affiliations:** 1 University of Pennsylvania, USA; 2 The University of North Carolina at Chapel Hill, USA; 3 UNC Project-Malawi, Malawi; 4 RTI International, USA; 5 Saint John of God College of Health Sciences, Malawi; 6 Psychiatry, University of KwaZulu-Natal, South Africa; 7 Psychiatry & Mental Health, Kamuzu University of Health Sciences, Malawi; 8 Government of Malawi Ministry of Health, Malawi; 9 Friendship Bench, Zimbabwe; 10 Kamuzu University of Health Sciences, Malawi

**Keywords:** suicide prevention, safety planning, adaptation, problem-solving, adolescents

## Abstract

There are few evidence-based suicide prevention interventions tailored for adolescents living with HIV (ALWH) in sub-Saharan Africa. The safety planning intervention targets acute suicidal behavior through co-creation of actionable coping strategies for use at the onset of suicide-related distress. We utilized the Assessment-Decision-Adaptation-Production-Topical Experts-Integration-Training–Testing framework to adapt and integrate safety planning into an existing Friendship Bench + peer support model for depressed ALWH in Malawi. We conducted interviews with ALWH who reported suicidal ideation or behaviors, their caregivers, healthcare facility leadership and police officers, and focus group discussions with healthcare facility staff, community and religious leaders and teachers in Lilongwe. The study team produced adapted manuals, sought and integrated expert topical feedback, trained interventionists using a training-of-trainers model and theater tested the protocol. Formative data yielded insights into acceptability, feasibility, delivery, content and implementation of safety planning. The final safety planning + Friendship Bench + peer support program consists of one safety planning session, five problem-solving sessions with suicide risk assessment and six peer support sessions. We revised the written Safety Plan to account for limited emergency services, modified the protocol for engaging guardians, integrated suicide assessment into the problem-solving sessions and incorporated suicide prevention activities into the peer support sessions.

## Impact statement

The core elements of safety planning can be adapted for different age groups, cultures and socio-economic contexts, recognizing the need for special considerations to account for psychiatric infrastructure in low-resource settings and vulnerabilities of adolescents living with HIV. In this manuscript, we describe how we used Assessment-Decision-Adaptation-Production-Topical Experts-Integration-Training–Testing (ADAPT-ITT) to adapt the safety planning intervention, feedback from the formative data collection and theater testing and the final adaptations to the intervention. Our application of ADAPT-ITT proved useful and allowed for a discussion of lessons for adapting safety planning in low-resource settings as well as the challenges and successes we encountered using ADAPT-ITT to guide the process.

## Introduction

Suicide among adolescents is a major global public health concern and remains the second leading cause of death among adolescents and young adults, with the majority of cases occurring in low- and middle-income countries (LMICs).

(Bantjes et al., 2016; Mokdad et al., 2016; Shain et al., 2016; WHO, 2016; Quarshie et al., 2020) In Malawi, the estimated past-year prevalence of suicidal among adolescents exceeds 10%. (Shaikh et al., 2016) However, data on suicidal ideations and behaviors (SIBs) in the country remain sparse. (MacLean et al., 2018; Malava, 2018; Pengpid and Peltzer, 2021) Among adolescents living with HIV (ALWH), an estimated 18–26% experience depression (Kim et al., 2015b), which is a well-established underlying risk factor for SIBs. (Kemigisha et al., 2019; Necho et al., 2021; Tsai et al., 2023) The risk of SIBs among ALWH is further heightened by social and structural challenges, including stigma and stress, inherent to living with HIV. (Shilubane et al., 2014; Adeyemo et al., 2019; Ashaba et al., 2019; Casale et al., 2019; Rukundo et al., 2020; Namuli et al., 2021; Kip et al., 2022; Gamassa, 2023) Research has shown that depressive symptoms and suicidal thinking resulting from stigma can manifest in refusal to take antiretroviral therapy (ART) and poor HIV care engagement as a passive form of suicidality (Kim et al., 2014; Kim et al., 2015a; Pantelic et al., 2018; Ashaba et al., 2019; Casale et al., 2019). Additionally, structural factors like the criminalization of suicide, contribute to stigma around SIBs in Malawi (Southern Africa Litigation Centre, 2022; Wu et al., 2022). However, in sub-Saharan Africa, there are few evidence-based suicide prevention interventions tailored for or tested among adolescents (Knettel et al., 2024) – let alone ALWH – for whom particular attention to developmental stage, effective family engagement and serostatus may be key (Abbott-Smith et al., 2023). There is an urgent need for culturally and contextually appropriate suicide prevention interventions that address acute and chronic suicidality and HIV care engagement among ALWH.

The Friendship Bench, a problem-solving therapy model for depression treatment, is increasingly being offered to adults (age 18 and older) in Malawi (Chibanda et al., 2011; Pierce, 2012; Udedi et al., 2019; Bengtson et al., 2023a; Pence et al., 2024). The Friendship Bench was recently adapted and enhanced to meet the needs of ALWH (Dao et al., 2025). The adapted and enhanced Friendship Bench utilizes young psychosocial counselors and peer supporters living with HIV to deliver problem-solving therapy and peer support sessions to improve mental health and HIV care engagement (Dao et al., 2025). While the Friendship Bench may support a reduction in suicidal thinking (Munetsi et al., 2018), it is not yet equipped to directly address SIBs among ALWH. Therefore, we chose to leverage the existing Friendship Bench + peer support (FB + PS) intervention and further enhance it with a specific evidence-based intervention for suicide prevention: safety planning. The safety planning intervention is a brief, evidence-based suicide prevention intervention that targets acute suicidal behavior through co-creation of a personalized list of coping strategies that can be used during onset or worsening of suicide-related distress (Stanley and Brown, 2012). Safety planning begins with the counselor eliciting from the client a narrative interview of a recent suicide crisis, allowing the counselor to demonstrate that acute risk of suicide dissipates over time (“the suicide risk curve”) and help the client identify “warning signs” – thoughts, feelings or behaviors – that suggest the client may be experiencing a crisis. The counselor will then work with the client to co-create a personalized, actionable safety plan, which includes six primary elements: (1) identifying warning signs of a suicide crisis; (2) internal coping strategies – activities that the client can engage in by themselves to distract them from their psychological pain; (3) identified positive supportive social environments that can distract from the current crisis; (4) trusted individuals who can be confided in and be supportive during a crisis; (5) contact information for healthcare services and other agencies that are equipped to manage SIBs; and (6) reducing access to lethal means (Stanley and Brown, 2012). Safety planning is the ideal method to enhance Friendship Bench to directly address SIBs among ALWH in Malawi, given it is one of the few suicide prevention interventions that has been adapted for adolescents in high-income countries (Albaum et al., 2025) has been used for adults in an African setting (Knettel et al., 2023a; Wagenaar et al., 2025; Wainberg et al., 2021) and can be delivered by non-specialists (Wainberg et al., 2021).

To guide the cultural adaptation of the safety planning intervention and integration with the FB + PS protocol, we used the Assessment-Decision-Adaptation-Production-Topical Experts-Integration-Training–Testing (ADAPT-ITT) framework (Wingood and DiClemente, 2008). ADAPT-ITT has been used to guide adaptations of evidence-based programs for people living with HIV (Wingood and DiClemente, 2008) and is an ideal model for adapting mental health interventions for ALWH in Malawi. This paper describes the application of the ADAPT-ITT framework to guide the adaptation and integration of safety planning for ALWH in Malawi into the FB + PS intervention. We present our process by ADAPT-ITT phase (including feedback from the formative qualitative research and theater testing) in the methods section and describe the final intervention adaptations. We then discuss relevant lessons for adapting safety planning in low-resource settings as well as the challenges and successes we encountered in the different phases of ADAPT-ITT.

## Methods

### Setting

This activity was conducted in Lilongwe, the capital city of Malawi, at three public primary care facilities that offer adolescent HIV care services. Research participants included ALWH aged 13–19 who reported SIBs, their guardians or caregivers, health care facility staff and leadership, police officers, teachers and community and religious leaders.

### Overview

Our multi-disciplinary team included both Malawian and US-based researchers, psychiatric specialists and representatives from the Malawi Ministry of Health. The team was co-led by an American global mental health and HIV researcher (based in Malawi) and a Malawian psychiatrist. The team included a Malawian clinical psychologist and a leader of a national psychiatric service provider as well as an American psychiatrist, an epidemiologist and a behavioral health researcher, all with expertise in implementing mental health and peer support interventions for adolescents in Malawi. Members of the Malawi Ministry’s noncommunicable disease and Mental Health department provided guidance on alignment with ministry priorities, feasibility and sustainability of the intervention. Together, we employed the eight stages of the ADAPT-ITT framework to adapt safety planning and develop the safety planning + Friendship Bench + peer support (SP + FB + PS) protocol (Table 1). We built on the existing youth-friendly FB + PS model, which had already been adapted for ALWH in Malawi using ADAPT-ITT (Dao et al., 2025). The existing FB + PS model included two protocolized manuals: one for the Friendship Bench problem-solving therapy sessions (delivered by trained psychosocial counselors) and one for the peer support sessions for improved mental health and HIV care engagement (delivered by peers living with HIV). Our new adaptation efforts focused heavily on adapting the safety planning intervention and integrating suicide prevention activities into the existing FB + PS model.

**Table 1.** Activities by ADAPT-ITT phase Table 1. long description.

Phase	Activity/Method
1. Assessment	Clinical expertise, prior research experience and literature review
2. Decision	Consideration to existing mental health infrastructure in Malawi, the suicide-related policy and legislative environment and review of existing suicide prevention evidence-base program
3. Adaptation	Interviews and focus group discussions with stakeholders (ALWH, caregivers, healthcare facility staff and leadership, police, teachers and community and religious leaders) Iterative adaptation workshops with previous youth Friendship Bench counselors and peer supporters living with HIV
4. Production	Production workshop to produce adapted manuals, review formative feedback and materials generated from the adaptation workshops
5. Topical experts	Developers of safety planning and Friendship Bench review manuals
6. Integration	Study team integrated feedback from topical experts; generates training materials
7. Training	Training-of-trainers model; trainers train interventionists with support from developer of the safety planning intervention
8. Testing	Theater test a mock safety planning session with youth community advisory board member and audience of key stakeholders

### Phase 1: Assessment

We assessed ALWH’s SIBs prevention and treatment needs through a literature review (as summarized in the introduction) and through discussion of our team’s vast prior research experience on adolescent mental health and HIV care in Malawi. Specifically, we reviewed published literature on SIBs among ALWH in Malawi and the sub-Saharan region to understand how and why ALWH experience SIBs, available prevention and treatment options, and unmet needs and discussed standard practices for addressing suicide risk as part of adolescent HIV care in Malawi. We conducted additional formative research as part of Phases 3 and 4 (described subsequently) that confirmed this assessment.

### Phase 2: Decision

Our multi-disciplinary team decided to adapt Safety Planning and integrate it into the existing FB + PS model for Malawian ALWH. This decision was made after iterative discussions taking into consideration existing mental health infrastructure in Malawi, the suicide-related policy and legislative environment (Nyasulu et al., 2024) and a review of existing suicide prevention evidence-based programs (Knettel et al., 2023b). Ultimately, we chose to adapt safety planning because it has been delivered as part of HIV care by non-specialists in the sub-Saharan region (Knettel et al., 2023a; Wainberg et al., 2021), can easily be incorporated into existing services and is increasingly being paired with other cognitive-behavioral therapies such as problem solving therapy to holistically address both acute suicide-risk and chronic SIBs (Beaudreau et al., 2023). We chose to leverage the existing FB + PS model, given (1) the recent adaptation for this specific context and population (Dao et al., 2025); (2) suggested feasibility of implementation (Udedi et al., 2018; Gaynes et al., 2021; Bengtson et al., 2023b; Pence et al., 2024) and (3) early evidence among adults suggesting the Friendship Bench may already support a reduction in suicidal ideation (Chibanda et al., 2016; Munetsi et al., 2018).

### Phase 3: Adaptation

Phase 3 included an iterative series of adaptation workshops and formative research. We held our first adaptation workshop in October 2024 with the previous adolescent Friendship Bench counselors, during which we discussed ALWH suicide prevention needs, the strengths and shortcomings of the Friench Bench for addressing suicide risk, the safety planning intervention, each step of the Safety Plan and the potential barriers to implementation. The Friendship Bench counselors drafted an initial translated Chichewa (the local language) Safety Plan that could be referenced in the interviews and focus group discussions.

We then conducted interviews with ALWH aged 13–19 who reported SIBs (n = 10) and their guardians or caregivers (n = 9) recruited from three public health facilities in Lilongwe (Table 2). ALWH were screened for SIBs using item 9 of the Patient Health Questionnaire-9 (PHQ-9) and Ask Suicide-Screening Questions (ASQ) Toolkit (Horowitz et al., 2012; Mutumba et al., 2014; Thompson et al., 2025), and suicide risk was assessed with the Suicide Risk Assessment Protocol (SRAP). (Landrum et al., 2023) All of these screening tools have been translated into Chichewa and reviewed by local psychiatric clinicians to ensure linguistic and cultural appropriateness. All ALWH (regardless of study enrolment) who reported SIBs received supportive counseling and follow-up from a nurse trained in suicide safety management. Of the 10 ALWH, nine allowed a caregiver to be interviewed. Caregivers included six parents, two sisters and one aunt.

**Table 2.** Formative study participants Table 2. long description.

N or mean (range)	ALWH	Caregivers	HCF staff	HCF leadership	Police	Community and religious leaders	Teachers
Type							
IDI	10	9		6	6		
FGD			3			3	1
Participants	10	9	18	6	6	18	6
Sex							
Female	4	8	15	4	4	8	3
Male	6	1	3	2	2	10	3
Average age	15.7 (13–18)	36.2 (21–46)	37.1 (24–56)	39.7 (35–46)	32.2 (26–42)	43.6 (21–72)	40.0 (23–58)

*Note*: FGD, focus group discussion; HCF, healthcare facility; IDI, in-depth interview.

We also conducted interviews with healthcare facility leadership (n = 6) and focus group discussions with healthcare providers (HCPs) (n = 3) at the three facilities. Healthcare facility leadership included nurses and clinicians in coordinator roles related to adolescent HIV care. HCPs included nurses, clinicians, HIV testing and counseling counselors, treatment supporters, a laboratory technician and a clerk.

Additionally, we conducted interviews with police officers (n = 6) and focus group discussions with teachers (n = 1) and community leaders (n = 1), and religious leaders (n = 2) from Lilongwe. These key stakeholders were included as all play key roles in suicide prevention and/or in the care of adolescents outside of clinical settings and may serve as sources of help in the face of mental health challenges given the scarcity of psychiatric services in Malawi. Furthermore, both the research team as well as our community advisory board felt these stakeholders would be well-positioned to share perspectives relevant to cultural acceptability and feasibility.

During the interviews and focus group discussions, interviewers described the initial safety planning session (suicide risk screening, narrative interview, co-creating a safety plan, engaging family, peer support, etc.), reviewed each step of the draft Chichewa Safety Plan and explained how ongoing suicide assessment would be integrated into the follow-up Friendship Bench sessions. Participants were asked to consider patient comfort, utility, examples of how each step on the safety plan could be completed, counselor and peer support preferences, and potential barriers and facilitators to delivery and engagement as well as to provide recommendations for adaptation. To assess linguistic and cultural validity of safety planning materials (suicide screener and written Safety Plan), ALWH participants were asked to reflect on the screeners (the experience of being, the acceptability of screening and to clarify their personal responses) and consider the language and acceptability of the written safety plan.

Interviewers conducted interviews and focus group discussions in Chichewa, which were recorded, translated and transcribed for analysis. The analysis was conducted using a thematic analysis approach (Guest et al., 2011). Summarized briefly, the team coded the transcripts using a thematic codebook and summarized themes relevant to participants’ reactions to the safety planning intervention, coping strategies for ALWH, support needs and preferences, provider and peer supporter preferences, availability of clinical or professional services, concerns about familial engagement, barriers to screening, implementation, follow-up, and sustainability and recommendations. Feedback related to acceptability and feasibility, delivery, content, implementation and the accompanying implications for adaptation is summarized in Table 3.

**Table 3.** Phase 3 formative feedback and implications for adaptation Table 3. long description.

Feedback and implications for adaptation	Quotes
Acceptability	
Positive reception of SP + FB + PS intervention Reinforce collaborative, patient-centered nature of SP + FB Ensure privacy, comfort and rapport with counselors and peer supporters Procedures for engaging trusted adults and guardians must alleviate concerns about disclosure of SIBs to caregivers and other adults and safeguard confidentiality	*“They will learn a lot of things that will help to keep them safe; they will learn tools and strategies to help them deal with suicidal thoughts and behaviors.” – Caregiver* *“My parents wouldn’t like that* [I’m in counseling]. *They will bring in their issues which will render the whole intervention meaningless. They wouldn’t like it because they would not know that what they do is what is affecting me.” – ALWH*
Delivery	
SP + FB counselors should be young, mature, empathetic, welcoming, kind, able to adapt to client needs and have a strong understanding of adolescent psychosocial and cognitive-behavioral functioning Peer supporters should be living with HIV, able to model healthy living, mature, knowledgeable and able to maintain confidentiality Interventionists should be aware of differences in SIBs and needs by age and gender Flexible delivery (weekend sessions, phone-based) could address structural barriers to engagement (financial hardship, transportation) and competing responsibilities (school, chores)	*“I think this person should not be judgmental; s/he should accept the client as she is. For example, he should not say ‘why would a good person like you think or do these things? Don’t you know this is a sin and you will die when you go to heaven? You are a problem the police should arrest you. You are a cancer to our society!’” – HCP* *“Intervention should not be gender biased, it needs to incorporate all genders and the program should try to understand what are the reasons that adolescents in certain category commit suicide.” - Teachers* **“** *For a person without the condition, I would be a little shy, I wouldn’t be as open, I would think ‘should I tell her about my condition? What for? If we disagree and argue in the future, she may badmouth me to her friends, before long, everyone will know about my situation.’” – ALWH*
Content	
Train interventionists to sensitively introduce the intervention and emphasize the lawful capacity of ALWH to disclose and discuss SIBs critical, given legal environment and stigma Capitalize on opportunity for ALWH to learn positive coping skills, receive psychoeducation on challenges specific to ALWH with SIBs (particularly stigma), share personal experiences and receive peer guidance on healthy coping strategies to improve effectiveness	“[Without clarification ALWH] *would wonder why they are being asked such question and would say to themselves, “are they asking me these questions so that when I say yes, they should get me arrested or do something to me.” – Caregiver* *“It means that the youths will be equipped with knowledge and information in many things because what they did not know, they are going to know through this mode of safety plan” – Community Leaders*
Implementation	
Caregiver involvement critical to lethal means restriction, but procedures for engagement must address challenges Reduce privacy risk posed by maintaining a written Safety Plan by removing explicit references to “suicide” Prepare for high workload and staff turnover to hamper maintaining adequate numbers of trained staff through integration with existing services (FB + PS) and use of the training-of-trainers model	*“I think there is a need of involvement of a guardian or someone who lives close with the young person in the same environment. If the young person would do this assessment with a health worker, we are not sure if those strategies* [to reduce access to lethal means] *will be implemented.” – HCP* *“Lack of or insufficient staff* [is the key challenge]*. We have very few counselors here, so, there is a need for additional staff.” –* HCP

Following the thematic analysis, we held two additional day-long adaptation workshops with previous Friendship Bench counselors and peer supporters living with HIV to discuss experiences delivering FB + PS relevant to suicide prevention (Dao et al., 2025), review the original Friendship Bench and peer support manuals, assess additions relevant to safety planning, symptom assessment and risk management, and generate new examples and story material.

### Phase 4: Production

The study team held a 2-day production workshop in May 2025 to formally generate background educational material specific to suicide, suicide risk management and safety planning, integrate the adapted safety planning procedures into the Friendship Bench manual, finalize the adapted Chichewa Safety Plan for the Malawian ALWH context and revise the peer support manual to include greater recognition of suicide risk and prevention among Malawian ALWH. We endeavored to ensure that all materials were culturally and linguistically tailored for Malawian adolescents. After discussing “standard of care” procedures and existing resources for managing suicide risk in Malawi, the team established procedures for supervision and referral for escalating suicide risk. The Malawi-based team debriefed with the US-based team each day to discuss progress and challenges.

### Phases 5 and 6: Topical experts and integration

The first draft of the combined SP + FB manual was reviewed by the entire research team, and then by external experts, including the developers of the original safety planning and Friendship Bench interventions who were chosen to ensure fidelity to the evidence-based components of both interventions.

### Phase 7: Training

We used a training-of-trainers model to train Malawian safety planning experts who could both train and supervise the psychosocial counselors. Attendees of the training included the local expert Friendship Bench trainer and study coordinator (SM), a psychiatrist (KK), a clinical psychologist (JJ), the director of a local mental health non-governmental organization (CM) and our ministry of health partners (MMU and JC). One of the developers of the original safety planning intervention (GB) led the training-of-trainers, which included 4 hours of didactics on the original safety planning intervention delivered over Zoom; two hybrid role-play sessions where trainees met in-person to role-play a safety planning session which GB evaluated over Zoom and a single in-person day devoted to evaluating sessions and providing feedback and supervisions to safety planning counselors.

The counselor training took place over 2 weeks. During week 1, the expert Friendship Bench trainer (SM) trained four psychosocial counselors on the Friendship Bench model. During week 2, GB joined two of the trained trainers (KK and SM), who led the safety planning training. Both weeks included a mix of didactics and role-playing, with feedback from the trainers and included discussion of identified delivery preferences, implementation barriers and legislative environment. Over the course of the training, counselors were both educated on and had an opportunity to discuss cultural explanatory models of distress, local beliefs about mental health and suicide and the broader sociocultural context in which ALWH live.

### Phase 8: Testing

The team theater tested a safety planning session. During the theater test, one trained counselor conducted a mock safety planning session with a member of the Youth Community Advisory Board (CAB) in front of an audience that included one CAB member, one ALWH, one caregiver, three FB counselors, two peer supporters, three nurses, one clinician, two research assistants and the District Health Office research committee chair. The mock session lasted approximately 1 hour. After completing the mock session, research staff facilitated a brief discussion with the audience members on their impressions of session content, length and counselor demeanor as well as recommendations for improvement. Audience members were asked to fill out a brief exit survey after each theater testing session with open-ended questions assessing the counselor’s performance, their likes/dislikes and suggested changes. Audience members appreciated the practicality, adaptability and collaborative nature of safety planning and the counselor’s ability to tailor the safety planning session based on the individual’s story, structure the session to meet the client’s specific needs and guide the client to identify their own individual coping strategies. The audience members praised the counselor for their tone, active listening and their attention to ensuring that the client fully understood every step of the safety plan. While audience members raised concerns about the session length, audience agreed that the counselor should devote sufficient time to fully address the client’s needs.

The feasibility and preliminary efficacy of the complete SP + FB + PS package will be tested in a pilot RCT (Stockton Stockton et al., 2025).

## Results

***Safety planning adaptations***

**Figure 1.**
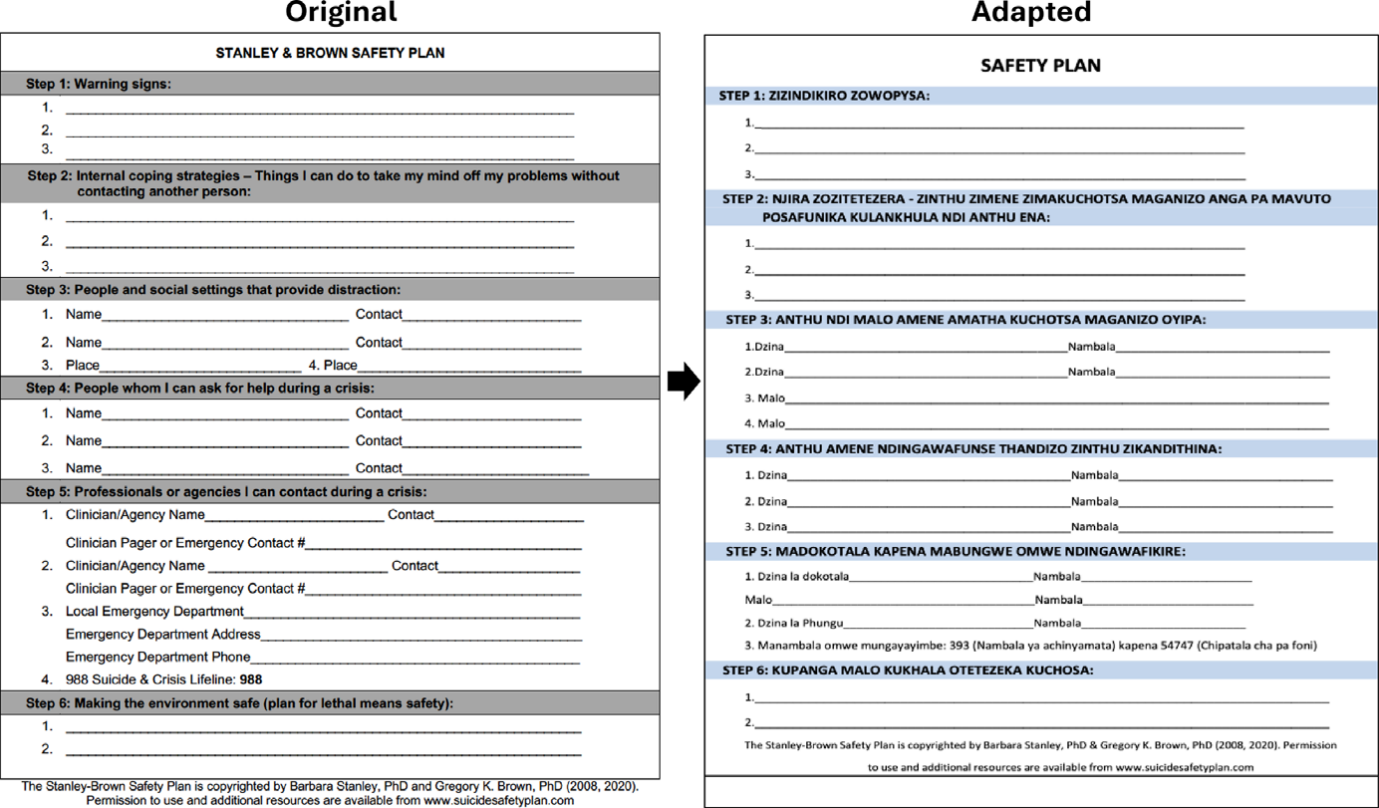
Adaptions to Written Safety Plan. Figure 1. long description.

We made adaptations to the written safety plan and the protocols for the narrative interview, identifying a trusted adult (Step 4), and caregiver engagement (generally and in Step 6 making the environment safer) (Table 4). First, we edited the written Safety Plan such that the word “suicide” was not written on the card to ensure that suicide risk would not be disclosed if the card was found (Figure 1). Second, we adapted the “Step 5: Professionals or agencies I can contact during a crisis,” considering limited psychiatric resources locally. Ultimately, this section contained the name of a doctor or nurse, two phone numbers and the location of the facility where the client seeks care, the name and number of the counselor and the numbers for the youth hotline and emergency hospital line.

**Table 4.** Overview of intervention adaptations Table 4. long description.

Intervention	Adaptations
Safety planning	Written Safety Plan Translation into Chichewa Ensure “suicide” not written on the physical copy of the Safety Plan “Step 5: Professionals or agencies I can contact during a crisis” includes the name of a doctor or nurse, two phone numbers and the location of the facility where the client seeks care; the name and number of the counselor and the numbers for the youth hotline and emergency hospital line Narrative Interview Adapt to focus on “suicidal crisis, episode or event” for those who are experiencing suicidal ideation and have not experienced an acute suicidal crisis Generate new probes for counselors to identify a discrete period of time when suicide risk was elevated and help demonstrate to the patient that suicide risk changes over time Identifying a Trusted Adult (Relevant to “Step 4: People whom I can ask for help during a crisis”) Added guidance on how to select a trusted adult from the Youth-Nominated Support Team for Suicidal Adolescents intervention package (King et al., 2006) Generated probes for adolescents to consider in identifying trusted adults Engaging caregivers in safety planning and in “Step 6: Making the environment safe” Added discussion after step six to identify an adult residing within the home that could be involved in safety planning and making the environment safe Protocolized how to engage that adult either in-person or over the phone and/or determine whether the adolescent can carry out the Safety Plan on their own
Friendship Bench	Content Reduced from 6 to 5 problem-solving therapy sessions Incorporate suicide risk assessment to each session Added discussion of safety plan utility, revisions and treatment engagement to each session
Peer support	Session 1 “Mental Health and HIV” Increased focus on suicide psychoeducation Include stories that incorporate an aspect of suicide risk Modified role-play activities to practice communicating about suicide risk Session 3 “ART adherence & Viral Load Testing” Added Undetectable = Untransmittable (U = U) information Session 5 “HIV Stigma” Added information on connection between HIV stigma and suicide risk Simplified activity by removing several superfluous group discussion prompts Session 6 “Planning for the Future” Incorporated suicide risk into stories Simplified activity for the younger adolescents

The original safety planning intervention included a narrative interview that provided foundational information for developing the safety plan. Specifically, the narrative interview helps individuals who have experienced an acute suicide crisis (e.g. previous attempt or hospitalization due to intent), understand the suicide risk curve, recognize how suicide risk dissipates over time and identify their own personal warning signs. To tailor safety planning for individuals who may not have experienced an acute suicidal crisis, but are reporting less severe suicidal ideation (e.g. suicidal thinking or intent, without a previous attempt or hospitalization due to intent), we expanded the narrative interview to focus on a recent “suicidal crisis, episode or event.” For individuals who had not experienced a crisis, we generated probes for the counselors to identify a discrete period of time when suicide risk was elevated and to help show the patient that suicide risk changes over time. These probes included questions about (1) what was happening at their worst point; (2) the events that occurred during their worst point, where they were, who they were with, etc. and (3) their thoughts and feelings and if they noticed any changes in their level of suicide risk over time. However, the adapted narrative interview maintained the therapeutic structure of the narrative interview in the original safety planning intervention, with the ultimate goal of helping clients be able to recognize warning signs and understand that the suicide risk wanes over time.

With respect to identifying a trusted adult, there was concern that adolescents would need additional guidance on how to select a trusted adult to confide in, given that this may require disclosure of HIV status or suicidal ideation or behaviors in a place where both HIV and suicide are highly stigmatized and attempting suicide is criminalized. Drawing from the Youth-Nominated Support Team for Suicidal Adolescents intervention package (King et al., 2006), counselors were given additional probes to help the adolescents identify caring adults from their family, school, or community, such as: “*Can you think of someone you might feel comfortable speaking to or people you think are easy to talk to”* or *“Perhaps someone who has not had much contact with you in the past, but who might be helpful now.”* Further, for each nominated adult, the counselor would ask the client to consider: *How do they know the person? Does the person know about their difficulties? How has this person helped them in the past? Are there any reasons this person may NOT be a good support person*, so that the counselor and client can together decide whether this individual is a suitable trusted adult?

The original safety planning intervention for youth assumes that the adolescent’s guardians are involved in safety planning from the onset and that guardians can help restrict access to lethal means and make the home environment safer. In the Malawian setting, guardians may not necessarily be at the facility when the client’s elevated risk of suicide is identified, be involved in their adolescent’s healthcare, or even reside in the same place as the adolescent. As such, we adapted the protocol for engaging a caregiver in “Step 6: Making the Environment Safer.” Namely, after completing “Step 6” on the safety plan, the counselor and client would discuss whether there is a caregiver or trusted adult who lives with the adolescent and could be involved in making the environment safer. If so, the counselor would either call that adult or invite that adult to attend the client’s next session. The counselor could then coach the trusted adult on implementing the safety plan, how to reduce access to lethal means in the home and make a plan to follow-up at a specified time with the client. If the client does not feel there is a trusted adult in the home who can be involved, the counselor will probe on the client’s perspective and situation to determine whether the client can carry out the plan on their own.

***Friendship Bench adaptation***

We decreased the number of Friendship Bench sessions to five, replacing one Friendship Bench session with the initial safety planning session. As a result, each client would receive six total counseling sessions (one safety planning + five Friendship Bench). The protocol for the Friendship Bench problem-solving therapy sessions remained the same, but suicide prevention follow-up activities were added to the beginning of each Friendship Bench session. Counselors would begin each Friendship Bench session with a suicide risk assessment with the PHQ-9, ASQ and SRAP to determine whether immediate escalation in psychiatric care is needed. The counselors would then facilitate a discussion around the utility of the safety plan, revisions to the safety plan (if needed) and treatment engagement and obstacles.

***Peer support adaptation***

We retained all six of the peer support sessions. We revised the Session 1 “Mental Health and HIV” to focus on suicide in addition to depression, include stories that incorporate suicide risk and role-play activities where participants practice communicating about suicide referrals. We added Undetectable = Untransmittable (U = U) language to Session 3 “ART adherence & Viral Load Testing.” We revised Session 5 “HIV Stigma” to include background information highlighting the connection between HIV stigma and suicide risk and simplified the session activity by removing several superfluous group discussion prompts. Finally, we revised Session 6 “Planning for the Future” so that the stories incorporated suicide risk and the session activity would be easier for the younger adolescents to complete. Sessions 2 and 4 on HIV status communication and HIV and sex remained the same.

## Discussion

We successfully used ADAPT-ITT to adapt safety planning for ALWH in Malawi, integrate safety planning into the existing FB + PS model and generate a comprehensive suicide prevention package complete with co-creation of a safety plan, problem-solving therapy and peer support. While safety planning is increasingly recognized as the “gold-standard” for suicide prevention (Stanley and Brown, 2012), adaptations to safety planning itself (such as integration with other cognitive-behavioral therapies) or to its implementation are warranted across patient populations and settings (Rogers et al., 2022). Our experience yielded the following adaptation recommendations related to fit for both acute and less severe suicidal experiences, choice of interventionists, translation, available profession or clinical support, and training on challenges to reducing access to lethal means. The minor adaptations to the narrative assessment probes described in the results may improve the utility of the safety planning session for both those who have attempted suicide as well as those who have only experienced suicidal ideation, although further research may be needed. We chose to train psychosocial counselors to deliver safety planning in response to both client preferences and available mental healthcare human resources. In settings where psychiatric resources are limited, there is potential for task-shifting suicide prevention services to non-psychiatric specialists and training lay counselors, community-based healthcare workers, or even school-based social support staff in safety planning (Knettel et al., 2023a; Wagenaar et al., 2025). Investment in developing and testing different delivery and supervision models for suicide prevention outside of the healthcare facility is lagging and would support low-resource expansion of mental healthcare (Nyasulu et al., 2024). In non-English speaking settings, we recommend a thorough forward-and-backward translation with review from both safety planning experts and local psychiatric experts to ensure both cultural and clinical relevancy. For Step 5 (Professional and Clinical Contacts), we recommend careful attention to the sources of professional or clinical support listed in the written safety plan, recognizing both healthcare system- and the patient-level resource limitations. For example, in Malawi, patients may not have a specific primary care provider, primary care facilities may not have accessible phone lines, and accessing care at a psychiatric facility, free emergency psychiatric services or suicide hotlines is challenging (Kauye, 2008; Nyasulu et al., 2024). In addition, patients may not have access to a phone with airtime to make calls for healthcare services. In generating an adapted safety plan, we recommend mapping available psychiatric services, listing locations and hours of healthcare facilities (along with or in lieu of provider phone numbers), and calling any identified toll-free healthcare hotlines for use in a crisis to ensure functionality. Furthermore, we recognize that limiting access to lethal means (Step 6) may be challenging in places where the patient population may commonly live in a single-room homes without lockable cabinets (or even across multiple single-room homes with extended family) and where the most common ways to die by suicide involve household items (kitchen knives, ropes, pesticides, etc.) (Dzamalala et al., 2006; Pengpid and Peltzer, 2021). Training the counselors to understand the landscape and challenges can help encourage appropriate probing and creative problem-solving to reduce access (such as keeping items out of sight). Similar adaptations to safety planning may be relevant for implementation in more rural high-income settings (Woodward et al., 2023) as well as LMICs.

Suicide prevention services tailored to the unique needs of people living with HIV are urgently needed. Given the dearth of suicide prevention interventions for people living with HIV (Kozlov et al., 2024), particularly in sub-Saharan Africa (Knettel et al., 2023b) and even more acutely for ALWH in the region (Knettel et al., 2024), we may not yet be able to ascertain the exact therapeutic components needed to support this vulnerable group. However, in Tanzania, a three-session suicide prevention intervention for adults living with HIV not only included gold-standard safety planning but also drew from evidence-based practices grounded in motivational interviewing, problem-solving, stigma reduction and action planning to support safety management and HIV care engagement (Knettel et al., 2025). In South Africa, a single-session intervention for adults newly diagnosed with HIV included psychoeducation on suicidal behavior, risk and protective factors, discussion on individual circumstances, HIV treatment and prevention, access to clinical services and advice on positive living (Govender et al., 2014). Our final intervention package includes many similar components, including safety planning, problem-solving and peer support with specific sessions designed to encourage engagement in HIV testing, provide psychoeducation on HIV and suicide, create space for discussing and addressing stigma and help adolescents plan for the future. Given the potential for suicidal behaviors to include reduced ART adherence (Kim et al., 2014; Kim et al., 2015a; Pantelic et al., 2018; Ashaba et al., 2019; Casale et al., 2019), suicide prevention services tailored for people living with HIV should also support sustained HIV care engagement.

Safety planning may need special modifications for adolescents, particularly those with chronic stigmatized conditions like HIV. For example, enhancing safety planning with additional cognitive-behavioral therapy, peer support or caregiver or family engagement may be particularly important for youth, given current evidence suggesting that the benefit of safety planning when delivered as a single-session intervention may be limited (Albaum et al., 2025). With respect to family engagement, caregiver involvement in safety planning proved both important and challenging to protocolize. In this setting, protocolizing how and when to engage caregivers must account for ALWH interacting with the healthcare system – particularly for HIV services – without their caregiver present, not living with their caregiver, caregivers who are unaware of their child’s HIV status and concerns about disclosing mental health concerns to family. An adaptation of safety planning for sexual and gender minority youth in the United States grappled with these same issues related to disclosure, and even generated specific caregiver-facing materials facilitate involvement (van der Star et al., 2025). While research suggests profound potential for parent or family involvement to be incorporated into safety planning and improve the efficacy of the intervention for adolescents (Wharff et al., 2019; Hill et al., 2020; Diamond et al., 2022; Albaum et al., 2025), further research into the timing and nature of familial involvement is warranted.

ADAPT-ITT proved to be a useful adaptation model, where the training and theater testing activities were particularly valuable.

ADAPT-ITT has previously been used to guide the adaptation of safety planning for emerging sexual and gender minority adults in a high-income setting, with differences in the activities included across the different phases (notably a lack of formative research and no theater testing) (Brown et al., 2023). We found the use of a training-of-trainers model and theater testing were paramount to our ADAPT-ITT process, and important tools for capacity building and community-engaged participatory research (Wingood and DiClemente, 2008; Mormina and Pinder, 2018; Merrill et al., 2025). The training-of-trainers activities took place concurrently with the formative data collection activities, which allowed the study team to receive advanced training on the “evidence-based” or “core” safety planning principles. Armed with this advanced training, the team could more effectively interrogate each component of safety planning, consider necessary adaptations and anticipate barriers and facilitators to delivery and implementation. For the trained counselors, the theater testing served as an opportunity to practice and receive feedback in more of a real-world setting, given that the role-play was conducted with a real adolescent without a background in safety planning. For audience members, the theater testing allowed the audience to rapidly develop familiarity with the intervention components. Conducting “classic” formative research to adapt an intervention can be challenging when participants are unfamiliar with the actual intervention components because it is difficult for participants to reflect on a hypothetical treatment model. However, providing participants with concrete materials to consider (e.g. a written Safety Plan) or the opportunity to view a session (e.g. theater testing), can facilitate discussions meaningful engagement. Furthermore, theater testing can also help establish buy-in from key stakeholders, from healthcare providers through district-level – or even ministry level – leaders who might not otherwise have an opportunity to understand the intervention or its utility. While theater testing does allow for reflection on content, it may fail to yield robust real-world implementation data (Warner and Pérez-Aronsson, 2025). When accompanied by formative research to assess service delivery preferences and support needs among the target population, other rapid-implementation methods – such as rapid-prototyping (Last et al., 2021) – may be useful in eliciting rapid pilot data on content, delivery and implementation.

### Limitations

The phases 1 and 2 (assessment and decision) activities could have been strengthened through structured procedures to assess and rank cultural relevance and feasibility and build consensus for decision-making. All formative research participants were recruited from the peri-urban population of Lilongwe District, and the feasibility of the SP + FB + PS intervention will be tested at peri-urban healthcare facilities, which are likely better resourced than more rural clinics that serve rural populations. Further research is warranted into the acceptability of safety planning and the validity of our suicide risk screening tools, as well as into whether additional adaptations are needed for delivery in other settings such as more rural healthcare systems, schools or even the police. To that end, the pilot RCT is ongoing and will yield greater insights into feasibility, acceptability, fidelity and barriers and facilitators to implementation in a more controlled research environment (e.g. delivered by trained psychosocial counselors and peer supporters employed by the research team) (Stockton Stockton et al., 2025). Should the SP + FB + PS intervention prove feasible in a research setting, we will need to draw from the field of implementation science to determine how best to scale-up, integrate and sustain evidence-based suicide prevention services into real-world adolescent HIV care (Boshe et al., 2023), given the prevailing resource and infrastructure limitations highlighted by our study participants.

## Conclusion

We used a robust process to adapt safety planning and integrate into FB + PS model. The resulting SP + FB + PS package may be added to the arsenal of evidence-based tools needed to support Malawi’s national suicide prevention response (Nyasulu et al., 2024). The experience yielded some key recommendations for how to apply ADAPT-ITT, adapt the safety planning intervention and deliver suicide prevention services that may be relevant regionally and in other low-resource settings.

## Data Availability

The de-identified data may be made available upon reasonable request.

## Abbreviations

ADAPT-ITT: Assessment-Decision-Adaptation-Production-Topical Experts-Integration-Training–Testing
ALWH: Adolescents living with HIV
FB: Friendship Bench
NHSRC: Malawi National Health Sciences Research Committee
PS: Peer support
RCT: Randomized controlled trial
SIBs: Suicidal ideation and behaviors
SP: Safety planning

## Table 1. Long description

The table has two columns: Phase and Activity or Method. From top to bottom, the phases and their activities are: 1. Assessment—clinical expertise, prior research experience and literature review. 2. Decision—consideration of existing mental health infrastructure in Malawi, suicide-related policy and legislative environment, and review of existing suicide prevention evidence-based programs. 3. Adaptation—interviews and focus group discussions with stakeholders including adolescents living with H I V, caregivers, healthcare facility staff and leadership, police, teachers, and community and religious leaders; iterative adaptation workshops with previous youth Friendship Bench counselors and peer supporters living with H I V. 4. Production—production workshop to create adapted manuals, review formative feedback, and materials from adaptation workshops. 5. Topical experts—developers of safety planning and Friendship Bench review manuals. 6. Integration—study team integrates feedback from topical experts and generates training materials. 7. Training—training-of-trainers model where trainers train interventionists with support from the developer of the safety planning intervention. 8. Testing—theater test of a mock safety planning session with a youth community advisory board member and an audience of key stakeholders.

Navigate back to Table 1..

## Table 2. Long description

From left to right, columns are labeled: N or mean (range), ALWH, caregivers, H C F staff, H C F leadership, police, community and religious leaders, teachers. The first row under ‘Type’ lists IDI and FGD. IDI counts: ALWH 10, caregivers 9, H C F leadership 6, police 6. FGD counts: H C F staff 3, community leaders 3, teachers 1. Participant totals: ALWH 10, caregivers 9, H C F staff 18, H C F leadership 6, police 6, community leaders 18, teachers 6. Female counts: ALWH 4, caregivers 8, H C F staff 15, H C F leadership 4, police 4, community leaders 8, teachers 3. Male counts: ALWH 6, caregivers 1, H C F staff 3, H C F leadership 2, police 2, community leaders 10, teachers 3. Average ages: ALWH 15.7 (13–18), caregivers 36.2 (21–46), H C F staff 37.1 (24–56), H C F leadership 39.7 (35–46), police 32.2 (26–42), community leaders 43.6 (21–72), teachers 40.0 (23–58).

Navigate back to Table 2..

## Table 3. Long description

Starting from the top, the table is divided into four main sections. The first section, Acceptability, spans both columns and includes feedback such as positive reception of S P plus F B plus P S intervention, reinforcing collaborative patient-centered care, ensuring privacy and rapport, and safeguarding confidentiality when engaging trusted adults. Quotes highlight perceived benefits and concerns about parental involvement and confidentiality. The second section, Delivery, also spans both columns and details desired counselor attributes—young, empathetic, adaptable, knowledgeable about adolescent functioning—and peer supporter qualities, including living with H I V and maintaining confidentiality. It emphasizes flexible delivery options and awareness of differences by age and gender. Quotes address the importance of nonjudgmental attitudes and gender inclusivity, as well as concerns about disclosure and stigma. The third section, Content, covers training interventionists to introduce the intervention sensitively, emphasizing lawful capacity for A L W H to disclose S I B s, and providing psychoeducation and peer guidance. Quotes reflect concerns about legal implications and the value of knowledge gained. The final section, Implementation, discusses caregiver involvement in lethal means restriction, privacy risks of written safety plans, staff workload and turnover, and integration with existing services using a training-of-trainers model. Quotes underscore the need for guardian involvement and highlight staff shortages as a challenge. Each section follows a top-to-bottom order, with bullet points in the left column and direct quotes in the right column.

Navigate back to Table 3..

## Table 4. Long description

The table has two columns labeled Intervention and Adaptations. From top to bottom, the first row lists Safety planning with adaptations including a written safety plan translated into Chichewa, omitting the word suicide on the plan, and detailed contact information for crisis steps. Narrative interview adaptations focus on suicidal crisis for those with ideation but no acute crisis, and new probes help identify periods of elevated risk. Guidance is added for identifying a trusted adult, with probes for adolescents and instructions from the Youth-Nominated Support Team for Suicidal Adolescents. Caregiver engagement is addressed by identifying an adult in the home for safety planning and specifying protocols for their involvement. The second row lists Friendship Bench with adaptations such as reducing problem-solving therapy sessions from six to five, adding suicide risk assessment and safety plan discussion to each session. The third row lists Peer support with adaptations by session: Session 1 increases suicide psychoeducation, includes stories on suicide risk, and modifies role-play for communication; Session 3 adds U equals U information; Session 5 adds content on HIV stigma and suicide risk and simplifies activities; Session 6 incorporates suicide risk into stories and simplifies activities for younger adolescents.

Navigate back to Table 4..

## Figure 1. Long description

The table is split vertically into two columns. The left column is titled ‘Original’ and contains the Stanley and Brown Safety Plan in English, divided into six steps: Step 1 is ‘Warning signs’ with three numbered blank lines; Step 2 is ‘Internal coping strategies’ with three blank lines; Step 3 is ‘People and social settings that provide distraction’ with spaces for four names or places and contacts; Step 4 is ‘People whom I can ask for help during a crisis’ with three name and contact spaces; Step 5 is ‘Professionals or agencies I can contact during a crisis’ with spaces for clinician or agency names, contacts, emergency departments, and the 988 Suicide and Crisis Lifeline; Step 6 is ‘Making the environment safe’ with a blank space for a plan. The right column is titled ‘Adapted’ and presents the same six-step structure, but the headings and prompts are translated into another language. Each step mirrors the structure of the original, with corresponding numbered lines and spaces for names, contacts, and places. The bottom of both columns contains copyright information and a website link.

Navigate back to Figure 1..
