# Supplementary material for: Integrating safety planning, problem-solving therapy and peer support for suicide prevention among adolescents living with HIV in Malawi: An application of the ADAPT-ITT adaptation framework
Source: Glob Ment Health (Camb). 2026 May 18;13:e118. doi: 10.1017/gmh.2026.10181 (PMC13279948; doi:10.1017/gmh.2026.10181)
Supplement: Peer review history

# Reviewer report: Review: Integrating safety planning, problem-solving therapy and peer support for suicide prevention among adolescents living with HIV in Malawi: An application of the ADAPT-ITT adaptation framework — R0/PR2

**DOI:** 10.1017/gmh.2026.10181.pr2

Suicide among adolescents is a global health crisis, is the second leading cause of death for adolescents and young adults,

occurs predominantly in low- and middle-income countries (LMICs)

The opening sentence highlights an important global concern; however, the phrasing could be improved for clarity and flow. I recommend rewording it to read more smoothly and to align with current WHO terminology. ‘’ Suicide among adolescents is a major global public health concern and remains the second leading cause of death among adolescents and young adults, with the majority of cases occurring in low- and middle-income countries (LMICs)

World Health Organization. Adolescent and young adult health [Fact sheet]. WHO; 26 Nov 2024. Available from: https://www.who.int/news-room/fact-sheets/detail/adolescents-health-risks-and-solutions

Gould M. Suicide and Suicide Attempts in Adolescents. Pediatrics. 2016;138(1):e20161420. doi:10.1542/peds.2016-142

In Malawi, the estimated past-year prevalence of thinking about suicide among adolescents is above

10%.(Shaikh et al. 2016) While data on suicidal ideation and behaviors (SIBs) in Malawi is sparse,(MacLean et al. 2018;

Malava 2018; Pengpid and Peltzer 2021) an estimated 18-26% of Malawian adolescents living with HIV (ALWH) are

affected by depression,(Kim et al. 2015a) an underlying risk factor for SIBs.(Kemigisha et al. 2019; Necho et al. 2021;

The paragraph presents important contextual data; however, the sentence structure and referencing need refinement for clarity and accuracy. The current version reads as a long, complex sentence with inconsistent citation formatting and an incomplete closing parenthesis. I recommend breaking it into shorter sentences and using consistent reference punctuation (e.g., “Shaikh et al., 2016; MacLean et al., 2018”). Additionally, it would strengthen the paragraph to clearly link depression to suicidal ideation and behaviors, since that connection is central to the argument. A suggested revision could be:

“In Malawi, the estimated past-year prevalence of suicidal thoughts among adolescents exceeds 10% (Shaikh et al., 2016). However, data on suicidal ideation and behaviors (SIBs) in the country remain sparse (MacLean et al., 2018; Malava, 2018; Pengpid & Peltzer, 2021). Among adolescents living with HIV (ALWH), an estimated 18–26% experience depression (Kim et al., 2015a), which is a well-established risk factor for suicidal ideation and behaviors (Kemigisha et al., 2019; Necho et al., 2021)

The Friendship Bench, a problem-solving therapy model for depression treatment increasingly used among adults inMalawi,(Bengtson et al. 2023b; Chibanda et al. 2011; Pence et al. 2024; Pierce 2012; Udedi et al. 2019) was recently

adapted and enhanced to meet the needs of ALWH.(Dao et al. 2025)

It is important that you double check your ethical tone and descriptions ‘’ Saying “used among adults” is correct but if the statement implies exclusively adults, you’d need to check what you mean by “adults” (e.g., 18+)

. Methods

Our multi-disciplinary team included both Malawian and US-based researchers, psychiatric specialists, and representatives from the Malawi Ministry of Health

consider clarifying the specific expertise or roles of each group (e.g., “Malawian and US-based researchers, including psychiatric specialists and Ministry of Health representatives”) to make the contribution of each partner clearer.

Glossary of Terms and Abbrreviations(Annex

Not sure about your word counts but i see value in uploading a table of the often used abbreviations

Our multi-disciplinary team included both Malawian and US-based researchers, psychiatric specialists, and

representatives from the Malawi Ministry of Health. Together, we employed the eight stages of the ADAPT-ITT

framework to adapt Safety Planning and develop the Safety Planning + Friendship Bench + Peer Support (SP+FB+PS)

protocol. (Table 1) We built on the existing youth-friendly FB+PS model, which had already been adapted for ALWH in

Malawi using ADAPT-ITT.(Dao et al. 2025) The existing FB+PS model included two protocolized manuals: one for the

psychosocial counsellor-delivered Friendship Bench problem-solving therapy sessions and one for the peer supporterdelivered peer support sessions. Our new adaptation efforts focused heavily on adapting the Safety Planning Intervention

and integrating suicide prevention activities into the existing FB+PS model.

Phase 1: Assessment

We assessed ALWH’s SIBs prevention and treatment needs through a literature review (as summarized in the

introduction) and through discussion of our team’s vast prior research experience on adolescent mental health and HIV

care in Malawi. We conducted additional formative research as part of Phase 3 & 4 (described below) that confirmed this

assessment.

The section presents a well-structured overview of the adaptation process and demonstrates a strong multidisciplinary collaboration. However, the use of the terms “peer support” and “psychosocial support” appears somewhat overlapping and may cause conceptual ambiguity. Since the Safety Planning intervention already contains psychosocial support components, it would be helpful if the authors could clarify what they specifically mean by “psychosocial support” in this context for example, what type or modality of support was provided, by whom, and how it differs from peer support within the integrated SP+FB+PS model.

We then conducted interviews with ALWH aged 13-19 who reported SIBs (n=10), their guardians or caregivers (n=9), and healthcare facility leadership (n=6) and focus group discussions with healthcare providers (HCPs) (n=3) recruited from three public health facilities in Lilongwe. (Table 2). ALWH were screened for SIBs using item 9 of the Patient Health Questionnaire-9 (PHQ-9) and Ask Suicide-Screening Questions (ASQ) Toolkit,(Horowitz et al. 2012; Mutumba et al. 2014; Thompson et al. 2025) and suicide risk was assessed with the Suicide Risk Assessment Protocol (SRAP).(Landrum et al. 2023) All ALWH (regardless of study enrolment) who reported SIBs received supportive counselling and follow-up from a nurse trained in suicide safety management. Of the 10 ALWH, nine allowed a caregiver to be interviewed. Caregivers were predominantly women, including five mothers, one father, two sisters, and one aunt. Healthcare facility leadership included nurses and clinicians in coordinator or leadership roles related to adolescent HIV care. HCPs included seven nurses, five clinicians, two HIV testing and counseling counselors, two treatment supporters, one laboratory technician and one clerk. Additionally, we conducted interviews with police officers (n=6) and focus group discussions with teachers (n=1) and community leaders (n=1), and religious leaders (n=2) from Lilongwe.

clarify recruitment and sampling logic – briefly explain why specific groups (e.g., police officers, teachers, and religious leaders) were included and how their perspectives contributed to the study’s objectives.

Distinguish between participant groups and data collection methods – for example, using separate sentences or subparagraphs for adolescents/caregivers, healthcare providers, and community stakeholders.

Simplify or tabulate participant characteristics – some information (e.g., caregiver composition, HCP roles) could be presented more clearly in Table 2 rather than in-text.

Ensure terminology consistency – it would be useful to specify whether all groups were interviewed or if some participated in focus groups only, as the mix of “interviews” and “FGDs” currently reads ambiguously

ADAPTATIONS

While the use of the ADAPT-ITT framework is commendable and demonstrates a strong application of adaptation science, the manuscript does not clearly articulate the cultural and linguistic adaptation processes undertaken. Cultural adaptation goes beyond procedural modification it requires consideration of language, idioms of distress, local beliefs about mental health and suicide, and the broader sociocultural context in which ALWH live.

I recommend that the authors elaborate on how the intervention was culturally and linguistically tailored for Malawian adolescents for instance, how key concepts were translated or localized, how cultural explanatory models of distress were integrated, and whether any steps were taken to ensure cultural validity (e.g., community consultations, pre-testing of translated tools).

RESULTS

The distinction between an acute suicide crisis and a recent suicidal episode or event needs clearer operational definitions. How did the authors determine thresholds for inclusion (e.g., what constitutes “less severe suicidal ideation”)?

It would be useful to explain whether the adapted “narrative assessment” maintained the original SPI’s therapeutic structure and risk monitoring components, or if it functioned primarily as an exploratory interview.

# Reviewer report: Review: Integrating safety planning, problem-solving therapy and peer support for suicide prevention among adolescents living with HIV in Malawi: An application of the ADAPT-ITT adaptation framework — R0/PR3

**DOI:** 10.1017/gmh.2026.10181.pr3

The authors are commended for a comprehensive adaptation of a suicide prevention plan for adolescents living with HIV. The manuscript is well written with methodological rigor following the ADAPT-ITT model.

A few minor comments below;

1. In the phase 1 assessment methodology, it should have a structured approach, i.e what areas were assessed, how were they ranked etc?

2. The methods section can start with the study setting, who the participants were before the overview.

3. In phase 5 and 6, Who were the external experts, how were they chosen? Was the Ministry of Health included for scaling up?

# Editor report: Recommendation: Integrating safety planning, problem-solving therapy and peer support for suicide prevention among adolescents living with HIV in Malawi: An application of the ADAPT-ITT adaptation framework — R0/PR4

**DOI:** 10.1017/gmh.2026.10181.pr4

**Affiliations:** University of South Florida, United States

Dear Dr. Stockton and Co-Authors, 

We have received reviews for your manuscript, with both reviewers recommending minor revisions. If you can address the reviewer comments thoroughly, we would be happy to consider a revised manuscript. Thank you for considering the special issue on Self-harm and Suicide: A Global Priority as an outlet for your work. 

Kristin Kosyluk

Guest Editor

# Editor report: Decision: Integrating safety planning, problem-solving therapy and peer support for suicide prevention among adolescents living with HIV in Malawi: An application of the ADAPT-ITT adaptation framework — R0/PR5

**DOI:** 10.1017/gmh.2026.10181.pr5

**Affiliations:** Social Work, University of South Florida, United States

No accompanying comment.

# Editor report: Recommendation: Integrating safety planning, problem-solving therapy and peer support for suicide prevention among adolescents living with HIV in Malawi: An application of the ADAPT-ITT adaptation framework — R1/PR7

**DOI:** 10.1017/gmh.2026.10181.pr7

**Affiliations:** University of South Florida, United States

Dear Dr. Stockton and Colleagues, 

I have had a chance to review your revised manuscript and response to reviewer comments. You did a nice job addressing reviewer comments, and we are pleased to accept your manuscript for publication in the special issue. Editorial staff will work with you to address some formatting problems, specifically with regard to in-text citations. Thank you for your important work. 

Best, 

Kristin Kosyluk

Guest Editor

# Editor report: Decision: Integrating safety planning, problem-solving therapy and peer support for suicide prevention among adolescents living with HIV in Malawi: An application of the ADAPT-ITT adaptation framework — R1/PR8

**DOI:** 10.1017/gmh.2026.10181.pr8

**Affiliations:** Social Work, University of South Florida, United States

No accompanying comment.
